# Patient-Reported Preference and Clinical Efficacy of Insulin Pen Devices With Safety Needles in Adolescents and Young Adults With Type 1 Diabetes: A Prospective Study

**DOI:** 10.7759/cureus.14555

**Published:** 2021-04-19

**Authors:** Ayman Al Hayek, Mohamed Al Dawish

**Affiliations:** 1 Department of Endocrinology and Diabetes, Prince Sultan Military Medical City, Riyadh, SAU

**Keywords:** type 1 diabetes, insulin pen therapy, safety pen needles, self-injection, preference, adherence

## Abstract

Purpose

Current evidence indicates that diabetic patients' preference and medication adherence can be affected by the type of insulin pen needles. We aimed to assess the impact of insulin pen devices with safety needles (SPN) on the usability, behavioral, lifestyle, and emotional aspects of type 1 diabetes mellitus (T1DM) in adolescents and young adults.

Patients and methods

We conducted a prospective single-center study on adolescent T1DM patients treated with multiple insulin doses using basal-bolus therapy for at least one year. Patients were followed for 12 weeks to compare the changes in the baseline usability and insulin fear of self-injection scales between SPN and conventional needles.

Results

In this 12-week study, we included 72 participants with a mean age of 15.5 ± 1.3 years. The mean disease duration was 5.1 ± 2.2 years. At 12 weeks, substantial improvement was evident in the SPN group, compared to the conventional group, in terms of the overall satisfaction score with a mean difference (MD) of 4.1 ± 1.9 (p < 0.01). Participants in the SPN group reported significant reduction in all aspect of fear from self-injection, such as being restless (MD = -1.4 ± 0.9), tense (MD = -1.8 ± 0.9), afraid (MD = -1.9 ± 0.9), worried (MD = -1.9 ± 0.9), nervous (MD = -1.7 ± 0.9), and brood using the SPN needles (MD = -1.6 ± 0.9), as compared to the conventional needles group. The glycemic control parameters, as determined by HbA1c and fasting blood glucose, exhibited significant improvements in the SPN group compared to the conventional group (p < 0.05).

Conclusion

SPN significantly improved usability, glycemic parameters, and reduced the fear of insulin self-injections amongst T1DM patients compared to conventional pen therapy.

## Introduction

Diabetes mellitus is an endemic metabolic disorder affecting individuals from various socioeconomic statuses and representing a global public health burden. Type 1 diabetes mellitus (T1DM) accounts for nearly 10% of the diabetes population, with a reported incidence of 15 per 100,000 people and a prevalence of 9.5% worldwide [1]. The pathogenesis of T1DM involves mainly immune-mediated destruction of pancreatic β-cells secondarily to complex interactions between genetic and environmental factors, such as infections, early introduction of cow’s milk, overweight, and psychological stress [2]. Subsequently, patients with T1DM are prone to a wide range of acute - such as diabetic ketoacidosis and cardiovascular ischemia - and chronic complications - such as retinopathy and nephropathy - as well as reduced life expectancy. Thus, life-long insulin therapy is the cornerstone in the management of T1DM patients, which can be administrated by subcutaneous injection or continuous infusion, if needed [3].

It is widely recognized that insulin delivery devices are crucial contributors to the effectiveness of insulin therapy and proper glycemic control. Initially, subcutaneous insulin injection was done via traditional syringes and vials, which remained the most popular methods for injection for nearly half a century [4]. Nonetheless, the use of traditional syringes was limited by being difficult to use, inaccurate dose preparation, negative psychological impacts, and high incidence of injection-related adverse events, such as swelling, pain, bleeding, and insulin leakage [5]. Since the mid-1980s, insulin pens have emerged as easy-to-use and reliable insulin delivery devices. The cumulative body of evidence highlights that insulin pens provide more convenient and reliable insulin delivery than traditional syringes. In return, insulin pens lead to better glycemic control, patients' satisfaction, treatment adherence, and long-term cost-effectiveness [4,6]. Over the recent decades, several new insulin pen needles were introduced to facilitate the injection, improve the delivery, minimize the pain, and reduce injection-related adverse events [7].

Nonetheless, the use of conventional insulin needles is not a complication-free procedure. Previous reports indicated that insulin needles accounted for most needlestick injuries in non-hospital settings [8]. Needlestick injuries and injection pain can trigger anxiety and fear from insulin injection, leading to potential disadvantages, such as limited adherence to insulin therapy, suboptimal glycemic control, and negative impact on the quality of life [9]. Therefore, several new needles were introduced to minimize the pain and reduce injection-related adverse events [7]. Safety pen needles (SPN), which were primarily introduced for hospital settings, can reduce accidental puncture risk through a retractable shield that automatically lockouts the needle after injection [10]. Recent reports highlighted that SPN was associated with ease-of-use, users' satisfaction, and a low incidence of needlestick injuries [11]. Moreover, it was shown that the use of SPN needed no additional education for the patients who self-administrated their insulin [12]. To date, few studies compared the difference in users' satisfaction and preference between SPN and conventional needles in the setting of T1DM.

Diabetes is an evolving health and economic burden in the Kingdom of Saudi Arabia (KSA); the KSA ranked as the eighth-highest country in the world with the number of people living with T1DM (35,000 children and adolescents) and came the fourth concerning the incidence of T1DM [13]. According to recently published literature, nearly half of the diabetic patients from KSA suffer from injection-related complications, such as bleeding and bruising [14]; it was noted that fear of self-injection is common among T1DM patients in KSA [15]. Therefore, it is imperative to study the impact of the type of insulin needles on T1DM patients' preference towards insulin pen devices in endemic countries like KSA. The present cohort study aimed to assess the impact of SPN on the usability, behavioral, lifestyle, and emotional aspects of T1DM patients, compared to the conventional needle.

## Materials and methods

The study protocol was approved by the Research and Ethics Committee of Prince Sultan Military Medical City (PSMMC), Riyadh, Saudi Arabia (IRB Ref No.#1279). Verbal and written informed consent forms were obtained from the guardians of the patients after clearly explaining the objectives and research methodology before completing the study measurement.

Study design and population

We conducted a prospective study on a convenience sample of 72 adolescents and young adult patients (age range 14-18 years) with T1DM through the period from November 2019 to March 2020 at the Diabetes Treatment Center, PSMMC Riyadh, Saudi Arabia. T1DM patients were recruited if they received multiple insulin doses using basal-bolus therapy for at least one year (>0.7 units per kg). Only patients who had never experienced insulin injection using a shorter needle than the 6 mm or had the experience to use any SPN to inject insulin medication were included. We excluded patients with impaired visual activity, disturbed cognitive functions, motor dysfunctions, tremors of a finger, or any other forms of medical instability that can interfere with insulin injection. Besides, patients with a history of mental disorders or cognitive impairment were excluded. We also ruled out the patients who were already under insulin pump treatment.

**Figure 1:**
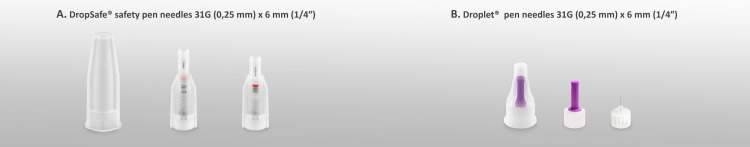
A graphical representation of the DropSafe® SPNs showing the (A) 31G (0.25 mm) x 6 mm DropSafe safety pen needles showing the shield and the outer cap; (B) 31G (0.25 mm) x 6 mm Droplet pen needles.

Eligible patients were shifted to SPN, which were provided with exactly the same length and gauge, 31G (0.25 mm) x 6 mm, of the conventional needles. The patients were educated on using DropSafe® SPNs (HTL-Strefa S.A., Poland) by the diabetes educator at enrollment. Then, all patients were followed up for 12 weeks to assess the changes in usability, fear of self-injection, and glycemic parameters. Patients did not receive any additional counseling on injection techniques during the study period (Figure 1).

Sample power estimation

With 72 patients, the study was able to detect an effect size of 0.35 standard deviations (SD) between baseline and the final visit using the paired t-test with a power of 80%, a confidence level of 5%, and an expected 10% drop-out rate.

Data collection

At baseline, the following parameters were collected from eligible patients as per the standard protocol of the center: demographic characteristics (age and gender), diabetes-related data (disease duration, type of the previous needle, total daily insulin dose, and the number of hypoglycemic events per month), weight, and glycemic parameters, including fasting blood glucose (FBG), glycated hemoglobin (HbA1c), and post-prandial glucose (PPG). The HbA1c was analyzed by using the COBAS INTEGRA 400 plus/800 analyzers (Roche Diagnostics, Indianapolis, IN, USA) at the hospital central laboratory. HbA1c <7% was selected as the cut-off value for proper glycemic control. Other variables related to injection were assessed, such as injecting site, reuse of needles, and the incidence of accidents while injecting.

All patients were assessed by a usability scale consisting of 14 questions related to six domains (pain and frightening judged from appearance of needles, installing and removing the needle, pain and sticking judged from inserting the needle, bleeding and bruising, dribbling of injected insulin, and power of pushing inserting button, and finally overall satisfaction) [16]. Scores for each question were obtained using a 10 points visual analog scale (VAS) with higher values indicating favorable answers. We used the Diabetes Fear of Injecting, a part of the Diabetes Fear of Injecting and Self-Testing Questionnaire (D-FISQ), to assess multiple domains, including the injector being restless, tense, afraid, worried, nervous, and brood about self-injecting himself/herself. Answers to each question were on a 4-point Likert Scale coded as 1 = never or almost never, 2 = sometimes, 3 = often, and 4 = almost always or always [17].

Patients were followed up for 12 weeks, and the follow-up appointments with the primary physician and diabetes educator were booked according to the protocol of the center. No further therapy or instructions were offered to the participants, who were asked to adhere to their usual insulin regimen with the standardized basal doses (dose, injection site, and injection time). At the end of the follow-up, all patients were assessed for the changes in body weight, glycemic parameters, usability scale, fear of insulin self-injection scale, injecting site, and reuse of needles. Hypoglycemia was defined as a blood glucose value ≤ 70 mg/dL.

Statistical analysis

The participants' demographics and other variables were summarized using mean ± SD for numeric variables and frequency distribution for categorical variables. For the duration of diabetes, the quartiles and range were also presented. The data normality was assessed by graphical presentation of the data and Kolmogorov-Smirnov test. Paired sample t-tests or Wilcoxon Signed Rank test, according to data normality, were used to assess the significance of the changes in the numerical variables. McNemar's test was used to assess changes in categorical variables. The reported distribution of injecting sites was also compared between the two groups using McNemar's test. A p-value of 0.05 or less was considered statistically significant. All analyses were done using IBM-SPSS (version 26, Armonk, NY, USA).

## Results

Demographic and clinical characteristics

We included 72 participants with a mean age of 15.5 ± 1.3 years. There was a slight male predominance (54.2%). The mean disease duration was 5.1 ± 2.2 years. Compared to the conventional needles, SPN was associated with substantially reduced total daily insulin dose, HbA1c%, FBG, PPG, and a monthly number of confirmed hypoglycemia when using SPN (Table 1).

**Table 1:** Diabetes-related variables. *Significant difference in results obtained using conventional to those using SPN. HbA1c: glycated hemoglobin; FBG: fasting blood glucose; PPG: post-prandial glucose; SPN: safety pen needle. .

	Conventional needle		Safety pen needle		SPN vs. conventional		
	Mean	SD	Mean	SD	Mean	SD	p-value
Weight (kg)	57.8	8.5	57.2	8.3	-0.6	2.0	0.011*
Total daily insulin dose (unit/kg/day)	0.9	0.2	0.8	0.2	-0.1	0.1	<0.01*
HbA1c (%)	8.4	0.9	8.0	0.6	-0.5	0.5	<0.01*
FBG (mg/dl)	197.9	24.2	182.6	18.3	-15.3	15.2	<0.01*
PPG (mg/dl)	196.1	19.6	178.1	18.1	-18.0	17.6	<0.01*
Confirmed hypoglycemia (per month)	2.5	1.1	1.1	0.8	-1.5	1.3	<0.01*

Self-reported usability

Participants reported an average significant increase in all aspects of the SPN needle's usability compared to the conventional one except the easiness to remove the needle, where no significant difference was reported. The patients' overall satisfaction score favors the SPN over the conventional needle, with a significant mean difference (4.1 ± 1.9; p < 0.01). Besides, participants reported a significant (p < 0.01) higher average favorable score when asked about frequency and intensity of pain using the SPN as compared to the conventional ones (Table 2).

**Table 2:** Comparing self-reported usability between conventional and SPN. †Higher values indicate more favorable answer in terms of usability (such as less pain and fear, less frequency of bleeding and bruising, etc.). *Significant difference in results obtained using conventional to those using SPN. SPN: safety pen needle.

	Conventional needle		SPN		SPN vs. conventional		
Usability of needle^†^	Mean	SD	Mean	SD	Mean	SD	p-value
Length of needle makes you think it is painful to insert	3.2	1.5	8.5	1.1	5.3	2.0	<0.01*
Length of needle makes you think it is frightening to insert	3.0	1.4	8.9	0.9	5.8	1.8	<0.01*
Thickness of the needle makes you think it is painful to insert	4.3	2.0	7.8	1.6	3.5	2.3	<0.01*
Thickness of the needle makes you think it is frightening to insert	3.3	1.6	7.8	1.5	4.5	2.5	<0.01*
Easy to install the needle into a pen-type insulin syringe	6.1	2.0	7.7	1.6	1.6	3.0	<0.01*
Easy to remove the needle from a pen-type insulin syringe	7.1	1.7	7.0	1.8	-0.1	2.5	0.853
Frequency of pain when inserting needle	3.8	1.9	8.7	1.0	4.9	2.1	<0.01*
Intensity of pain when inserting needle	3.8	1.9	8.7	0.9	4.9	2.0	<0.01*
Frequency of feeling sticking judged from inserting the needle	4.0	1.7	8.5	1.3	4.5	2.3	<0.01*
Frequency of bleeding at the site of injection	5.6	2.0	7.8	1.8	2.2	2.3	<0.01*
Bruising after injection	5.1	2.1	7.6	1.7	2.6	2.6	<0.01*
Dribbling of injected insulin from cutaneous tissue	4.5	1.8	6.7	2.0	2.3	2.8	<0.01*
Intensity of handling power of pushing inserting button	5.3	1.8	6.4	1.7	1.1	2.1	<0.01*
Overall satisfaction	4.7	1.7	8.8	0.9	4.1	1.9	<0.01*

Self-reported fear of self-injecting

In terms of fear of self-injection, participants reported significant decreases in all aspect of fear such as being restless (MD = -1.4 ± 0.9), tense (MD = -1.8 ± 0.9), afraid (MD = -1.9 ± 0.9), worried (MD = -1.9 ± 0.9), nervous (MD = -1.7 ± 0.9), and brood using the SPN (MD= -1.6 ± 0.9), as compared to the conventional ones (Table 3).

**Table 3:** Self-reported fear of self-injection. ^†^Higher values indicate more fear. *Significant difference in results obtained using conventional to those using SPN. SPN: safety pen needle.

	Conventional needle		SPN		SPN vs. conventional		
Insulin fear of self-injection^†^	Mean	SD	Mean	SD	Mean	SD	p-value
Restless	2.8	0.7	1.4	0.5	-1.4	0.9	<0.01*
Tense	3.1	0.8	1.2	0.4	-1.8	0.9	<0.01*
Afraid	3.2	0.7	1.3	0.5	-1.9	0.9	<0.01*
Worried	3.2	0.7	1.3	0.5	-1.9	0.9	<0.01*
Nervous	2.9	0.7	1.3	0.5	-1.7	0.9	<0.01*
Brood	2.9	0.8	1.2	0.4	-1.6	1.0	<0.01*

Other outcomes related to usability

In terms of using a needle more than one time, 33.3% of the patients used the conventional needle more than one time compared to 0% in the SPN. Moreover, 43.1% of the participants reported at least one accident injury in the past four weeks while using the conventional needles, as compared to 0% on the SPN (p < 0.01; Table 4).

**Table 4:** Other reported outcomes related to usability. *Significant difference in results obtained using conventional to those using SPN. ^‡^21 (29.2%) uses the needle twice and 3 (4.2%) use the needle 3 times. SPN: safety pen needle.

Variables		Conventional needle		SPN		
		n	%	n	%	p-value
Use needle more than one time	No	48	66.7	72	100	<0.01*
	Yes	24^‡^	33.3	0	0	
History of needle stick accident in the past 4 weeks	None	41	56.9	72	100	<0.01*
	yes	31	43.1	0	0	
Patient rotates his/her injection site	No	39	54.2	6	8.3	<0.01*
	Yes	33	45.8	66	91.7	

Finally, reported injection sites were significantly different by participants while using conventional needles compared to the SPN (p < 0.01). In particular, when using conventional needles, 8.3%, 20.8%, 20.8%, and 50.0% of the participants reported abdomen only, thigh only, arm, and multiple injection sites, respectively. When participants were using the SPN, 90.0% reported multiple injection sites while the rest reported abdomen only.

## Discussion

Since its discovery more than one hundred years ago, insulin remains the safest and most potent glucose-lowering therapy. Over the years, insulin treatment has undergone dramatic changes due to the development of more insulin analogs and new insulin delivery devices, including pumps, pens, and safety pen needles [4]. Therefore, it is vital to assess the difference between these various delivery devices and how they can assist patients, in the future, with respect to diabetes management and satisfaction [18]. Despite the potential advantages of SPN over conventional needles, a limited number of clinical studies compared the difference in patients' preference between SPN and conventional needles. In the present study, we assessed the impact of changing the type of needles from conventional ones to SPN on T1DM patients' perceptions of satisfaction, fear, and glycemic control. Our findings favored the SPN over the conventional needle in simplicity, usability, and patient's satisfaction. Moreover, it was associated with less pain and fear from self-injection. Patients' utilization of SPN was associated with improved glycemic control.

Conventional insulin injection methods are available in various sizes, providing up to 100 units. Nevertheless, they are less satisfactory, require a multi-step preparation, in addition to special storage and sufficient vision during dose infusion, to determine the needed units [19]. Insulin pens are more acceptable, light-weight, have a larger scale that facilitates dosages, and provide an audible "click" for each unit, which helps to overcome many visual issues [20]. Nonetheless, needles-related injuries can increase patients' anxiety and fear of injecting, leading to poor treatment adherence [8]. Previous reports showed that fear of self-injection is common in adolescents with T1DM and it can lead to non-compliance with treatment regimen [15]. SPN can reduce accidental puncture risk through a retractable shield that automatically lockouts the needle after injection; once the injection site is pressured, the sliding sleeve encircling the needle is retracted and, thus, prevent needle-stick injuries [10]. The advantages of insulin pens over conventional methods are well-characterized in the literature. However, to our knowledge, only a few studies assessed the preference towards SPN in non-hospital settings. In this prospective study, we demonstrated that the SPN significantly improved the patients' preference towards the insulin pen therapy three months after its use, compared to conventional needles. The SPN achieved more favorable scores regarding the perception of insertion, ease-of-use, frequency of insertion-associated pain, bleeding at the site of injection, bruising, and dribbling. The SANITHY study compared SPN and conventional syringes regarding direct and indirect costs, safety, and patients' satisfaction [21]. Their findings demonstrated that about 94.7% of the patients found SPN as a convenient method, 85% reported that it was safe and simple, 87.2% think that SPN might help in maintaining a good glycemic control, 89.3% would recommend SPN to other peoples with diabetes, and 74.2% will continue taking insulin at home using SPN. These findings were even much higher in the subgroup of newly insulin users, which might result in high compliance, as the high rate of patients' satisfaction may enhance their adherence to treatment [22]. Among a group of trained healthcare workers, Adams and Elliott showed that the staff was highly satisfied with the usability, safety, and compatibility of the SPN. Moreover, they have suggested that using SPN with sufficient training might reduce injection-related adverse events [23]. Likewise, Veronesi et al. [24], highlighted that the compatibility and simplicity of SPN contributed to a high satisfaction (85%) among the general hospital nurses in northern Italy. Our findings also run in parallel with a recent report by Malinowski et al., in which the SPN was associated with ease-of-use, user satisfaction, and a low incidence of needlestick injuries [11].

The patients' perception towards an insulin delivery device can have a notable impact on their glycemic control level; it was reported that pain and fear perception could limit patients' adherence and lead to poor infection technique, resulting in suboptimal delivery of the insulin dose [25]. Such observations were confirmed by previous reports, in which poor glycemic control (defined as HbA1C >7%) was significantly associated with higher degree of fear from self-injection amongst adolescents with T1DM [26]. Thus, insulin delivery devices with less pain and fear can presumably lead to better glycemic control. This hypothesis was evident by our findings, in which the use of SPN was associated with significant reductions in the HbA1c, FBG, and PPBG. Luijf et al. [27], found no significant difference between SPN and conventional methods in terms of HbA1c; however, a significant reduction in the FBG was observed in the SPN group compared to the conventional group (p = 0.003).

There are many safety concerns linked to the complexity of insulin administration with traditional insulin delivery devices. For example, it was found that needle characteristics, such as needle length, were significantly associated with a higher incidence of lipohypertrophy in young patients with T1DM [28]. Many authors suggested an increased risk of inaccurate insulin dose drawn with the conventional syringes than SPN [29]. The results of Ramadan et al. [30], demonstrated that pen needles led to a lower incidence of hypoglycemic episodes than conventional syringes, which might be correlated to inaccuracy in insulin dosing. Our study showed that the risk of developing hypoglycemia is substantially lower with SPN compared with a conventional needle (MD= -1.5 ± 1.3; p < 0.01). Moreover, no patient-reported needle stick accident when using SPN compared with 31 patients in the conventional needle. We assumed that the lower incidence of needlestick injuries with SPN may stem from the mechanical characteristics of the SPN itself, SPN is loaded with a retractable shield that prevents needle exposure during and after injection [10]. However, Bossi et al. [21], showed that there was no significant difference between the SPN and conventional syringes with regards to the events of in-hospital hypo- and hyperglycemia (p = 0.3). 

To the best of our knowledge, this is the first study in the MENA region that assessed the T1DM patients' preference towards the SPN, while most of the published studies have been conducted to evaluate nurses' perceptions and satisfaction with the use of SPN. Nevertheless, we acknowledge that our study has some limitations, including the small sample size and the study conducted in a single center. It can be overcome by conducting studies on a larger scale. The study’s population was also limited to adolescents and the results cannot be generalized to older population. We recommend that future studies should explore the impact of using SPN on various populations, such as patients with complications and elderly. Another limitation is the lack of controlled approaches to control the impact of various confounders on the plausible relation between the use of SPNs and glycemic outcomes. Despite the limitations, the present work gives valuable data on the positive improvements seen in adolescents and young adult patients with T1DM concerning the level of satisfaction and fear from the self-injection, the lower number of hypoglycemic episodes, and improvement in the glycemic parameters after 12 weeks of using the SPN. Future studies are recommended to compare various SPNs and their impact on patients’ preferences.

## Conclusions

This prospective study's findings reveal that patients with T1DM can experience improvement in the level of insulin needles usability, HbA1c values, and hypoglycemia after a 12-week usage of the SPN. The SPN significantly improved usability and reduced the fear of insulin self-injections amongst T1DM patients compared to conventional pen therapy. However, further studies are crucial so that the long-term and consistent use of the SPN can confirm that the patient satisfaction levels among the T1DM patients are enhanced.

## References

[1] Mobasseri M, Shirmohammadi M, Amiri T, Vahed N, Hosseini Fard H, Ghojazadeh M (2020). Prevalence and incidence of type 1 diabetes in the world: a systematic review and meta-analysis. Health Promot Perspect.

[2] DiMeglio LA, Evans-Molina C, Oram RA (2018). Type 1 diabetes. Lancet.

[3] Silver B, Ramaiya K, Andrew SB (2018). EADSG Guidelines: Insulin Therapy in Diabetes. Diabetes Ther.

[4] Kesavadev J, Saboo B, Krishna MB, Krishnan G (2020). Evolution of insulin delivery devices: from syringes, pens, and pumps to DIY artificial pancreas. Diabetes Ther.

[5] Guo X, Wang W (2017). Challenges and recent advances in the subcutaneous delivery of insulin. Expert Opin Drug Deliv.

[6] Singh R, Samuel C, Jacob JJ (2018). A comparison of insulin pen devices and disposable plastic syringes - simplicity, safety, convenience and cost differences. Eur Endocrinol.

[7] Frid AH, Kreugel G, Grassi G (2016). New insulin delivery recommendations. Mayo Clin Proc.

[8] Kiss P, De Meester M, Braeckman L (2008). Needlestick injuries in nursing homes: the prominent role of insulin pens. Infect Control Hosp Epidemiol.

[9] Rubin RR, Peyrot M, Kruger DF, Travis LB (2009). Barriers to insulin injection therapy: patient and health care provider perspectives. Diabetes Educ.

[10] Yu CH (2012). Cases: "safety" technology: a hidden cause of diabetic ketoacidosis. CMAJ.

[11] Malinowski M, Serafin A, Prazmowska-Wilanowska A (2021). DropSafe safety pen needle helps to prevent accidental needlesticks after injections: results of a simulated clinical study. J Infect Prev.

[12] Gillespie E, Canning E (2014). Introducing insulin pen needle safety devices in Australia to protect nurses. Aust Nurs Midwifery J.

[13] Robert AA, Al-Dawish A, Mujammami M, Dawish MAA (2018). Type 1 diabetes mellitus in Saudi Arabia: a soaring epidemic. Int J Pediatr.

[14] Alhazmi GA, Balubaid RN, Sajiny S, Alsabbah R (2020). Assessment of insulin injection technique among diabetic patients in Makkah region in Saudi Arabia. Cureus.

[15] Al Hayek AA, Robert AA, Babli S, Almonea K, Al Dawish MA (2017). Fear of self-injecting and self-testing and the related risk factors in adolescents with type 1 diabetes: a cross-sectional study. Diabetes Ther.

[16] Miyakoshi M, Kamoi K, Iwanaga M, Hoshiyama A, Yamada A (2007). Comparison of patient's preference, pain perception, and usability between Micro Fine Plus 31-gauge needle and Microtapered NanoPass 33-gauge needle for insulin therapy. J Diabetes Sci Technol.

[17] Mollema ED, Snoek FJ, Pouwer F, Heine RJ, van der Ploeg HM (2000). Diabetes Fear of Injecting and Self-Testing Questionnaire: a psychometric evaluation. Diabetes Care.

[18] Snell-Bergeon JK (2015). Assessing insulin delivery device satisfaction in patients with type 1 and type 2 diabetes. Diabetes Technol Ther.

[19] Marín-Peñalver JJ, Martín-Timón I, Sevillano-Collantes C, Del Cañizo-Gómez FJ (2016). Update on the treatment of type 2 diabetes mellitus. World J Diabetes.

[20] Hyllested-Winge J, Sparre T, Pedersen LK (2016). NovoPen Echo(®) insulin delivery device. Med Devices (Auckl).

[21] Bossi AC, Veronesi G, Poerio CS (2016). A prospective study for introducing insulin pens and safety needles in a hospital setting. The SANITHY study. Curr Diabetes Rev.

[22] Davies MJ, Gagliardino JJ, Gray LJ, Khunti K, Mohan V, Hughes R (2013). Real-world factors affecting adherence to insulin therapy in patients with Type 1 or Type 2 diabetes mellitus: a systematic review. Diabet Med.

[23] Adams D, Elliott TS (2006). Impact of safety needle devices on occupationally acquired needlestick injuries: a four-year prospective study. J Hosp Infect.

[24] Veronesi G, Poerio CS, Braus A (2015). Determinants of nurse satisfaction using insulin pen devices with safety needles: an exploratory factor analysis. Clin Diabetes Endocrinol.

[25] Fu AZ, Qiu Y, Radican L (2009). Impact of fear of insulin or fear of injection on treatment outcomes of patients with diabetes. Curr Med Res Opin.

[26] Berlin I, Bisserbe JC, Eiber R, Balssa N, Sachon C, Bosquet F, Grimaldi A (1997). Phobic symptoms, particularly the fear of blood and injury, are associated with poor glycemic control in type I diabetic adults. Diabetes Care.

[27] Luijf YM, DeVries JH (2010). Dosing accuracy of insulin pens versus conventional syringes and vials. Diabetes Technol Ther.

[28] Al Hayek AA, Robert AA, Braham RB, Al Dawish MA (2016). Frequency of lipohypertrophy and associated risk factors in young patients with type 1 diabetes: a cross-sectional Study. Diabetes Ther.

[29] Church TJ, Haines ST (2016). Treatment approach to patients with severe insulin resistance. Clin Diabetes.

[30] Ramadan WH, Khreis NA, Kabbara WK (2015). Simplicity, safety, and acceptability of insulin pen use versus the conventional vial/syringe device in patients with type 1 and type 2 diabetes mellitus in Lebanon. Patient Prefer Adherence.

